# On the Suitability of Phosphonate-Containing Polyamidoamines as Cotton Flame Retardants

**DOI:** 10.3390/polym15081869

**Published:** 2023-04-13

**Authors:** Alessandro Beduini, Domenico Albanese, Federico Carosio, Amedea Manfredi, Elisabetta Ranucci, Paolo Ferruti, Jenny Alongi

**Affiliations:** 1 Dipartimento di Chimica, Università Degli Studi di Milano, Via C. Golgi 19, 20133 Milan, Italy; 2 Dipartimento di Scienza Applicata e Tecnologia, Politecnico di Torino, Alessandria Campus, Via T. Michel 5, 15121 Alessandria, Italy

**Keywords:** phosphonate-containing polyamidoamines, intumescent flame retardants, functional coatings, cotton

## Abstract

A novel polyamidoamine (M-PCASS) bearing a disulfide group and two phosphonate groups per repeat unit was obtained by reacting *N*,*N*′-methylenebisacrylamide with a purposely designed bis-sec-amine monomer, namely, tetraethyl(((disulfanediylbis(ethane-2,1-diyl))bis(azanediyl))bis(ethane-2,1-diyl))bis(phosphonate) (PCASS). The aim was to ascertain whether the introduction of phosphonate groups, well-known for inducing cotton charring in the repeat unit of a disulfide-containing PAA, increased its already remarkable flame retardant efficacy for cotton. The performance of M-PCASS was evaluated by different combustion tests, choosing M-CYSS, a polyamidoamine containing a disulfide group but no phosphonate groups, as a benchmark. In horizontal flame spread tests (HFSTs), M-PCASS was a more effective flame retardant than M-CYSS at lower add-ons with no afterglow. In vertical flame spread tests, the only effect was afterglow suppression with no self-extinguishment even at add-ons higher than in HFSTs. In oxygen-consumption cone calorimetry tests, M-PCASS decreased the heat release rate peak of cotton by 16%, the CO_2_ emission by 50%, and the smoke release by 83%, leaving a 10% residue to be compared with a negligible residue for untreated cotton. Overall, the set of results obtained envisage that the newly synthesized phosphonate-containing PAA M-PCASS may be suitable for specific applications as flame retardant, where smoke suppression or reduction of total gas released is a key requirement.

## 1. Introduction

Cellulosic materials are among the most flammable materials for domestic use [1,2]. Regarding cotton, they are also widely employed as reinforcement in composites in different technical sectors, including the automotive and aerospace industries and construction [3]. Not surprisingly, cellulosic materials constitute a frequent trigger point for fires that occur in inhabited environments. In fact, the Center of Fire Statistics of International Association of Fire and Rescue Services in the latest bulletin containing fire statistics of 48 countries, representing 3.3 bln inhabitants, reported 4.0 million fires and 20.8 thousand civilian fire deaths in 2020 [4].

Impregnation with solutions of flame retardant (FR) substances is a common practice to overcome this drawback [5]. Historically, phosphorus-based compounds such as alkyl phosphates [6], polyphosphoric acid derivatives [7], phytic acid, and phytic acid derivatives [8,9,10,11,12] have been and still are among the most popular and effective cotton FRs. Among them, tetrakis (hydroxymethyl) phosphonium salts (Proban^®^ by Solvay Group, Bruxelles, Belgium) and *N*-methyloldialkyl phosphonopropionamides (Pyrovatex^®^ by Huntsman, TX, USA) have been the main FRs for cotton used over the past 40 years [6,13]. However, they have significant environmental impact due to the release of formaldehyde during both manufacturing and service life [6], hence international regulations require more eco-compatible and non-toxic cotton FRs [14]. It has also been reported that several phosphonates are endowed with a remarkable efficacy as flame retardants for cotton [6], thanks to their ability to promote the dehydration of cellulose rather than depolymerization, thus increasing the amount of char formed at high temperatures [15].

Polyamidoamines (PAAs) are a family of highly versatile multifunctional polymers obtained, in linear form, by the aza-Michael polyaddition of *prim*-monoamines, including naturally occurring α-amino acids or bis-sec-amines with bisacrylamides. PAAs are structurally highly versatile, can be engineered to be biodegradable and biocompatible, and, in the past, have mainly been investigated as potential biomedical polymers [16,17]. In the last decade, many PAAs were proven to be remarkably effective FRs for cotton [18,19,20]. In particular, the PAAs deriving from the polyaddition of *N*,*N*′-methylenebisacrylamide (M) with glycine (M-GLY) and most other natural α-amino acids were particularly effective in this regard [21]. As a rule, some PAAs proved capable of extinguishing the flame in horizontal flame tests (HFSTs) with add-ons as low as 4.5% [18], but, in most cases, failed to extinguish the flame in the more stringent Vertical Flame Tests (VFSTs), even at add-ons of 20% or more. However, the PAA obtained from the polyaddition of *L*-cystine with *N*,*N*′-methylenebisacrylamide (M-CYSS) effectively extinguished the flame in VFSTs as well [20]. This behavior was ascribed to the release of sulfur-containing volatiles acting as radical scavengers capable of quenching the flame. However, rather disappointingly, in HFSTs, it was significantly less effective than other previously tested non-sulfur PAAs, such as M-GLY [18].

Given these premises, in this work, it was thought to be worth studying whether, similarly to disulfide groups, the addition of phosphonate groups in PAA repeat units could increase their efficacy as FR for cotton by synergistically improving the intumescence of PAAs and favoring cotton charring.

To this purpose, a novel PAA was prepared and tested, namely, the aza-Michael polyaddition products of M with tetraethyl(((disulfanediylbis(ethane-2,1-diyl))bis(azanediyl))bis(ethane-2,1-diyl))bis(phosphonate) (PCASS), henceforth referred to as M-PCASS, whose repeat unit contained four phosphonic ester groups and one disulfide group. In this work, the effectiveness of M-PCASS as FR for cotton was investigated by both HFSTs and VFSTs, as well as oxygen-consumption cone calorimetry tests. The aim of this paper is to report on the results obtained.

## 2. Materials and Methods

### 2.1. Materials

*N*,*N*′-methylenebisacrylamide (M, 99%), L-cystine (CYSS, 99%), lithium hydroxide monohydrate (LiOH∙H_2_O, 98%), 1 M hydrochloric acid (HCl), cystamine dihydrochloride (CASS, >98%), triethylamine (TEA, >99%), diethyl ether, dichloromethane, and anhydrous sodium sulfate (Na_2_SO_4_) were supplied by Sigma-Aldrich (Milano, Italy). Diethyl vinylphosphonate (97%) was supplied by TCI Chemicals (Israel) and used as received. Cotton (COT) with an area density of 240 g∙m^−2^ was purchased from Fratelli Ballesio S.r.l. (Torino, Italy).

### 2.2. Methods

Thermogravimetric analyses (TGA) of PAAs and PAA-treated cotton fabrics were performed in inert (nitrogen) and oxidative (air) atmosphere on a TAQ500 thermogravimetric balance from 50 to 800 °C at a heating rate of 10 °C∙min^−1^ (TA Waters, Milano, Italy); samples (circa 5 mg) were placed in open alumina crucibles, in either inert or oxidative atmospheres, under 20 mL∙min^−1^ gas flow.

The surface of untreated and PAA-treated cotton fabrics was analyzed by an EVO 15 equipped with a ULTIM MAX 40 probe scanning electron microscope (SEM) (Zeiss, Ramsey, NJ, USA) operating at 8.5 mm working distance, under a 5 kV beam voltage, equipped with Energy-Dispersive X-ray Spectroscopy (EDX, Jena, Germany) to perform elemental analyses. A fabric piece (5 mm × 5 mm) was fixed to a sample holder and then gold metallized.

### 2.3. Synthesis of Tetraethyl(((disul-fanediylbis(ethane-2,1-diyl))bis(azanediyl))bis(ethane-2,1-diyl))bis(phosphonate) (PCASS)

**Scheme 1:**
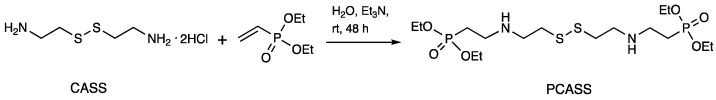
Synthesis of tetraethyl(((disulfanediylbis(ethane-2,1-diyl))bis(azanediyl))bis(ethane-2,1-diyl))bis(phosphonate) (PCASS).

Tetraethyl(((disulfanediylbis(ethane-2,1-diyl))bis(azanediyl))bis(ethane-2,1-diyl))bis(phosphonate) (PCASS) was synthesized, optimizing the procedure previously reported [22]. In brief, in a one-necked round-bottom flask, cystamine dihydrochloride (0.46 g; 2.00 mmol) was dissolved in H_2_O (2 mL) and, then, keeping the solution in an ice bath, triethylamine (0.56 mL; 4.01 mmol) was slowly added. The mixture was warmed up to room temperature, stirred for 15 min, then a solution of diethyl vinyl phosphonate (0.64 mL; 4.01 mmol) in H_2_O (2 mL) was added. Once the addition was finished, the reaction mixture was left under stirring at room temperature for 48 h. The solution was treated with diethyl ether (5 mL), and the product was extracted from the aqueous one with dichloromethane (2 × 5 mL) after phase separation. The combined organic phases were dried over Na_2_SO_4_, filtered, and the solvent removed under vacuum to give a pale-yellow oil. Yield: 0.69 g, 73%. The reaction scheme is shown in Scheme 1. The structure of PCASS was confirmed by ^1^H-and ^31^P-NMR (Figures S1 and S2 in Supplementary Materials).

### 2.4. Synthesis of Polyamidoamines

**Scheme 2:**
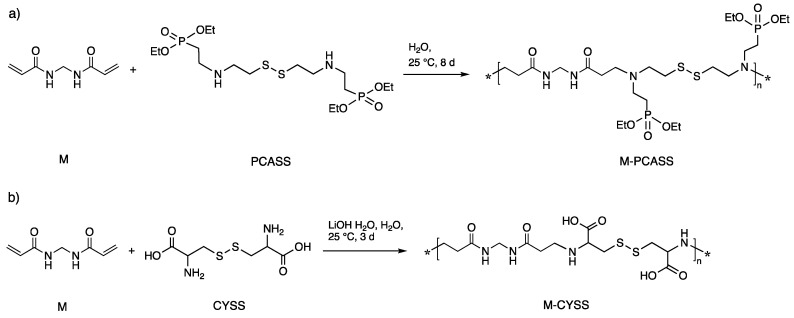
Synthesis of M-PCASS (**a**) and M-CYSS (**b**).

**Synthesis of M-PCASS.** *N*,*N*′-methylenebisacrylamide, M, (0.50 g; 3.20 mmol) and PCASS (1.54 g; 3.20 mmol) were dissolved in water (3.00 mL) at 45 °C. After complete monomer dissolution, the reaction mixture was cooled to room temperature and gently magnetically stirred at room temperature for 8 days in the dark. Then, it was diluted with H_2_O (30 mL) and the pH adjusted to 4.0 with 1 M HCl. The product was finally retrieved by freeze-drying. The yield was nearly quantitative. The reaction scheme is shown in Scheme 2a.

**Synthesis of M-CYSS.** M-CYSS was synthesized following the procedure already reported [20]. In brief, *N*,*N*′-methylenebisacrylamide (1.56 g, 10.00 mmol), L-cystine (2.40 g, 10.00 mmol), and lithium hydroxide monohydrate (0.86 g, 20.00 mmol) were dissolved in H_2_O (7 mL) and heated under stirring to 45 °C until the complete dissolution of the monomers occurred. The reaction mixture was cooled to 25 °C and left at this temperature for 3 days in the dark, then it was diluted to 70 mL with water, and the pH was adjusted to 9.2 with a 1 M HCl solution. The product was finally retrieved by freeze-drying. The yield was nearly quantitative. The reaction scheme is shown in Scheme 2b.

The structure of M-PCASS and M-CYSS was confirmed by ^1^H-NMR and FT-IR/ATR spectroscopy (Figures S3–S6, respectively).

### 2.5. Treatment of Cotton Fabrics with PAAs

Strips of cotton fabric of a 30 mm × 60 mm size were dried by heating at 100 °C for 2 min and then weighed. Subsequently, they were impregnated twice with aqueous solutions of PAAs of suitable concentration, drying for 3 min at 100 °C after each deposition. The total dry solid add-ons (*Add-on*, wt.%) were determined by weighing each sample before (*W_i_*) and after drying following impregnation (*W_f_*). The add-ons were calculated according to Equation (1):

(1) $$Add-on=\frac{W_{f}-W_{i}}{W_{i}}\times 100$$

The concentrations of the impregnating PAA solutions and the final add-ons were: 4.0 wt.% for 8.0% add-on and 3.0 wt.% for 6.0% add-on.

Treated-cotton fabrics were coded with the prefix COT/followed by the code of the PAA employed. Therefore, COT/M-PCASS stands for cotton samples treated with M-PCASS and COT/M-CYSS for cotton samples treated with M-CYSS.

### 2.6. Combustion Tests of PAA-Treated Cotton Fabrics

Horizontal flame spread tests (HFSTs) and Vertical flame spread tests (VFSTs) were carried out by applying a 20 ± 5 mm long butane flame to the short side of 30 mm × 60 mm specimens according to the ISO 3795 [23] and ISO 15025 [24] standards, modified in terms of cotton size specimens and flame application time. In the horizontal configuration, the sample was positioned in a metallic frame tilted at an angle of 45° along its longer axis and then ignited for 3 s. In the vertical configuration, the butane flame was applied for 2 s on the center of the short side of the specimens. All combustion tests were tripled, and the total combustion time (s) and residual mass fraction (RMF, %) were assessed.

The resistance to a 35 kW∙m^−2^ irradiative heat flux of square fabric samples (100 mm × 100 mm) was investigated using an oxygen-consuming cone calorimeter (Nose-lab ATS advanced, Milan, Italy). Measurements were carried out in a horizontal configuration following a procedure previously reported [25], optimized based on the ISO 5660 standard [26]. Parameters such as the time to ignition (TTI, s), heat release rate peak (pkHRR, kW∙m^−2^), total heat release (THR, MJ∙m^−2^), and residual mass fraction (RMF, wt.%) were determined [27]. Total Smoke Release (TSR, m^2^∙m^−2^), carbon monoxide (CO), and carbon dioxide (CO_2_) yields, both expressed in kg^−1^, were also determined. Prior to the combustion tests, all specimens were conditioned to constant weight at 23 ± 1 °C for 48 h at 50% relative humidity in a climatic chamber. Each experiment was performed in triplicate and the standard deviation calculated.

## 3. Results and Discussion

### 3.1. Synthesis of PAAs

In this work, the efficacy of the PAA coded M-PCASS (Scheme 2a) as a flame retardant for cotton was investigated and compared to that of the PAA coded M-CYSS (Scheme 2b), which had already been shown to suppress cotton combustion in vertical flame tests [20]. Both M-PCASS and M-CYSS contain a disulfide group per repeat unit. Additionally, M-PCASS contains two phosphonate groups, whereas M-CYSS only contains two carboxylate groups. Therefore, in this respect, M-CYSS represented a useful benchmark to test the suitability of introducing phosphonate groups into PAA repeat units to enhance their efficacy as flame retardants.

M-CYSS was synthesized by the aza-Michael polyaddition of *N*,*N*′-methylenebisacrylamide with *L*-cystine, as already reported (Scheme 2b) [20], where M-PCASS was obtained from the reaction of *N*,*N*′-methylenebisacrylamide with the purposely synthesized monomer tetraethyl(((disulfanediylbis(ethane-2,1-diyl))bis(azanediyl)) bis(ethane-2,1-diyl))bis(phosphonate) (PCASS) monomer (Scheme 2a). PCASS was synthesized optimizing a previously reported procedure [22]. In fact, by modifying the purification procedure, in particular, by using dichloromethane as the second extraction solvent, the reaction yield significantly increased with respect to the value reported in the reference literature (from 27% to 73%).

Both M-PCASS and M-CYSS were obtained following standard synthetic procedures normally adopted in PAA synthesis [16]. They both took place in a single step in water at pH 11 and with an overall solid concentration of 40% by weight. In both cases, the reaction mixtures gradually became homogeneous. After being left under occasional stirring at 25 °C for 8 days (M-PCASS) and 3 days (M-CYSS), the polymeric products were recovered by freeze-drying and used without further purification. Their structures were evaluated by ^1^H-NMR and FT-IR/ATR spectroscopies (Figures S3–S6, respectively) and their number-average molecular weights calculated from the ^1^H-NMR spectra from the ratio between the integrals of the resonance peaks relative to the terminal units and the integrals of the resonance peaks relative to the internal repeat units (see for details the caption to Figure S3). While the spectra of M-PCASS were consistent with low molecular weight products, specifically, decamers, those of M-CYSS indicated a high molecular weight polymer.

### 3.2. Thermal Stability of PAAs

**Figure 1:**
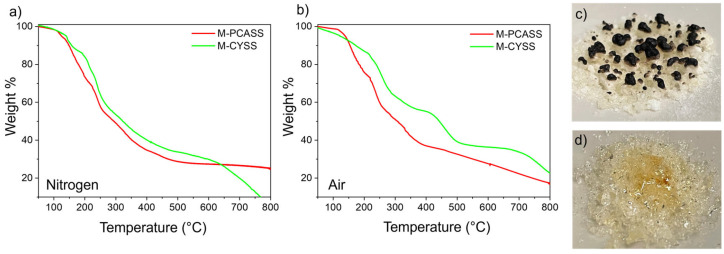
TG thermograms of M-PCASS and M-CYSS in: (**a**) nitrogen and (**b**) air; residues of the ignition test on: (**c**) M-CYSS and (**d**) M-PCASS.

The TG thermograms of M-PCASS in nitrogen and in air in the 50–800 °C range were compared with those of M-CYSS under the same conditions (Figure 1a,b). The onset decomposition temperature at 10% weight loss T_onset10%_, the temperature at maximum weight loss rate T_max_, and the residual mass fraction at 800 °C RMF_800_ are reported in Table 1. The weight loss curves of both polymers showed similar multimodal patterns both in nitrogen and in air. In nitrogen, the T_onset10%_ of both polymers were only slightly different, being 149 °C for M-PCASS and 157 °C for M-CYSS. Between 200 and 600 °C, M-CYSS was slightly more stable than M-PCASS, but its thermal decomposition was apparently much faster above 650 °C, reaching RMF = 0 at around 770 °C, whereas M-PCASS left 25% RMF at 800 °C. By comparing the TG curves in air, two different multi-step paths were observed. M-CYSS was clearly more stable than M-PCASS, although both left a significant RMF at 800 °C (23% and 17%, respectively).

The higher thermal stability of M-PCASS and M-CYSS in air, with respect to that observed in nitrogen, was ascribed to their ability to form an intumescent and thermally stable char, as already observed for other PAAs [18,19,20,28]. This behavior of both PAAs was further confirmed by ignition tests (Figure 1c,d), where PAA powders were exposed to a butane flame for 10 s. Neither polymer ignited, and both left a final residue equal to 99% of the original weight. However, they reacted differently to the flame impingement: M-CYSS decomposed superficially, forming an expanded carbonaceous crust (Figure 1c), whereas M-PCASS melted and formed a protecting film, leaving the underlying powder apparently intact (Figure 1d).

### 3.3. FT-IR and Morphological Characterization of PAA-Treated Cotton Fabrics

Untreated and PAA-treated cotton fabrics (COT, COT/M-PCASS and COT/M-CYSS, respectively) were characterized by FT-IR/ATR spectroscopy (Figure S7). All spectra revealed diagnostic bands of cellulose; both PAA-treated cotton fabrics showed the typical absorption bands of PAAs and, particularly in the spectra of COT/M-PCASS, the absorption bands of the phosphonate groups were observed. In fact, COT/M-PCASS showed the following signals: 3330 (ν O–H), 2860 (ν CH_2_), 1375 (δ C–H), 1320 (δ O–H), and 1015 cm^−1^ (ν C–O) for cellulose; 1651 (ν C=O) and 1540 cm^−1^ (δ N–H) for PAA; and 1215 (ν P=O) and 790 cm^−1^ (ν P–C) for the phosphonate group.

**Figure 2:**
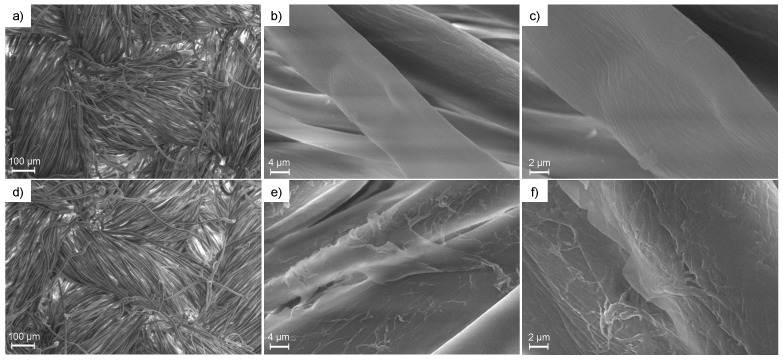
SEM micrographs at different magnifications of: (**a**–**c**) untreated cotton (250×, 5000× and 10,000×) and (**d**–**f**) cotton fabrics treated with 8% add-on M-PCASS (250×, 5000× and 10,000×).

The morphologies of the COT/M-PCASS samples were analyzed by SEM (Figure 2d–f) and compared to that of untreated cotton (Figure 2a–c). The surfaces of the cotton fibers were mostly smooth in both untreated and M-PCASS-treated cotton. Furthermore, the high-magnification SEM micrographs of the COT/M-PCASS sample revealed the presence of a thin layer of M-PCASS interconnecting the cotton fibers (Figure 2e,f).

**Figure 3:**
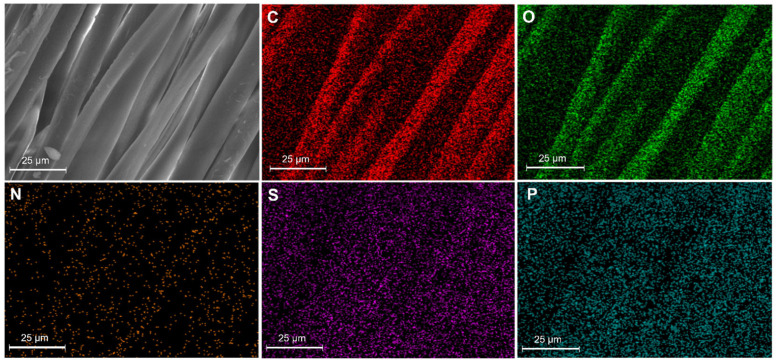
EDX analysis of COT/M-PCASS. Distribution of carbon (C), oxygen (O), nitrogen (N), sulfur (S) and phosphorus (P).

The EDX analysis of COT/M-PCASS (Figure 3) revealed a homogeneous distribution not only of carbon (C) and oxygen (O) but also nitrogen (N), sulfur (S), and phosphorous (P) present in M-PCASS.

### 3.4. Thermal Characterization of PAA-Treated Cotton Fabrics

**Figure 4:**
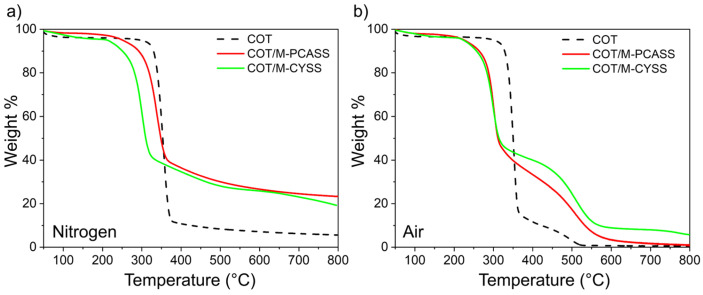
TG thermograms of COT/M-PCASS and COT/M-CYSS in: (**a**) nitrogen and (**b**) air.

The thermal stability of untreated and PAA-treated cotton fabrics was investigated by TG analysis in nitrogen and air in the range 50–800 °C (Figure 4). T_onset10%_, T_max_, and RMF values are reported in Table 2. In nitrogen (Figure 4a), a single main weight loss was observed for all samples. Untreated cotton was thermally stable up to 300 °C, with approximately 80% of the weight loss occurring in the range of 300–375 °C. M-PCASS- and M-CYSS-treated cotton fabrics anticipated cotton degradation, as clearly demonstrated by the T_onset10%_ and T_max_ values (Table 2). However, compared to untreated cotton, the COT/PAA samples formed a significantly higher amount of thermally stable char above 350 °C, with RMF values almost constant until 800 °C. At this temperature, the RMF values were indeed 29 and 23% for COT/M-PCASS and COT/M-CYSS (Table 2).

As widely reported elsewhere [29], the TG curve of untreated cotton in air showed two main weight losses, from 300 °C to 375 °C and from 375 to 515 °C (Figure 4b). Moreover, as in nitrogen (Figure 4a), it is thermally stable up to 300 °C, losing 80% of its weight above this temperature. However, its RMF dropped to negligible levels due to thermal oxidation as early as around 550 °C.

In the cases of the PAA-treated cotton fabrics, the TG curves of COT/M-PCASS and COT/M-CYSS are almost superimposable up to 300 °C and did not significantly differ in the remaining temperature range, showing three different decomposition steps, namely, in the intervals 210–320 °C, 320–440 °C, and 440–600 °C. As reported for all PAAs thus far tested [18], they anticipated cotton degradation, reducing both T_onset10%_ and T_max_ values, forming in the meantime a higher amount of char between 300 and 550 °C. Above 550 °C, the RMF reduced slowly down to non-negligible values at 800 °C (Table 2).

### 3.5. Combustion Tests of PAA-Treated Cotton Fabrics

As above stated, the aim of this work was to study the flame-retardant efficacy of disulfide-based PAAs containing additional phosphonate groups, to determine whether synergism was established between these two types of functional groups. To this aim, the combustion behavior of COT/M-PCASS was investigated by Horizontal Flame Spread Tests (HFSTs), Vertical Flame Spread Tests (VFSTs), and oxygen-consumption cone calorimetry tests. The results were compared not only with those of COT but also of COT/M-CYSS, whose performance in this respect had already been studied [20].

#### 3.5.1. Horizontal Flame Spread Tests

**Figure 5:**
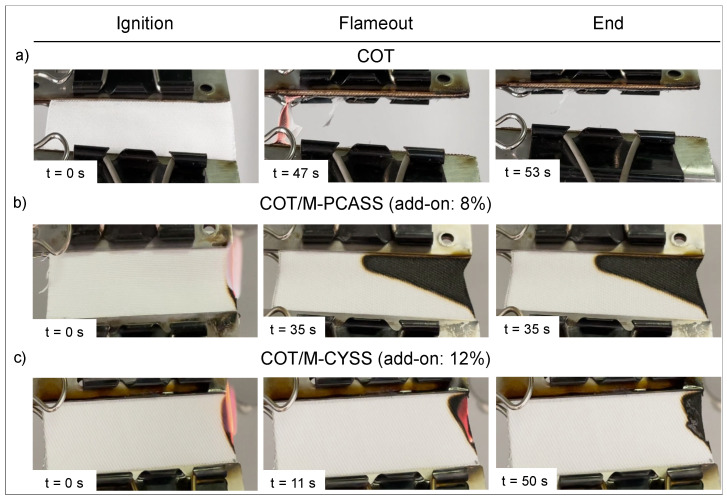
Snapshots of untreated cotton (**a**) and cotton treated with M-PCASS (**b**) and M-CYSS (**c**) at the ignition, flameout, and extinguishment steps during HFSTs.

COT and PAA-treated cotton fabrics were tested in a horizontal configuration, applying a butane flame for 3 s. Table 3 shows the main combustion data obtained. Figure 5 shows some snapshots of COT, COT/M-PCASS, and COT/M-CYSS taken during HFSTs. When the flame was applied to cotton, it vigorously and completely burned, leaving <1% residual mass fraction (RMF) (Figure 5a). PAA-treated cotton specimens were tested at different add-ons to find the minimum add-on for extinguishing the flame (Table 3).

COT/M-PCASS suppressed the flame at an 8% add-on, leaving an RMF of 81%, whereas COT/M-CYSS blocked the flame only at an add-on above 12%, leaving an RMF of 93%, (Table 3, Figure 5b and Figure 5c, respectively). On the other hand, it has already been shown that the M-CYSS coating failed to achieve self-extinguishment at an 8% add-on [20]. Moreover, COT/M-PCASS extinguished the flame, showing no signs of afterglow, while COT/M-CYSS exhibited a significant afterglow that partially consumed the fabric after the flameout.

**Figure 6:**
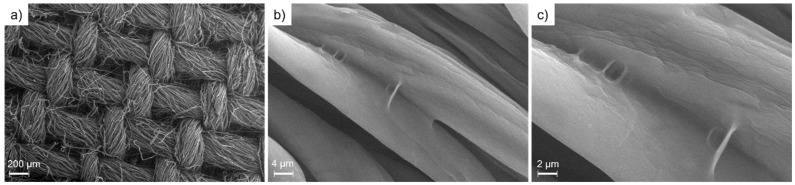
SEM micrographs of COT/M-PCASS residue left by HFSTs at different magnifications: (**a**) 100×, (**b**) 5000×, and (**c**) 10,000×.

The morphology of the burnt area of COT/M-PCASS with an 8% add-on was observed by SEM; Figure 6 shows the surface of the residue after the combustion test at three different magnifications (Figure 6a–c). The burnt areas of the fabrics maintained the original texture of cotton, and the fibers were intact even after combustion. The efficacy of the coating and its ability to protect cotton is apparent from Figure 6b,c, from which it can be seen how the M-PACSS coatings, by melting, weld the cotton fibers together. This is an agreement with the behavior observed in ignition tests (Figure 1d).

**Figure 7:**
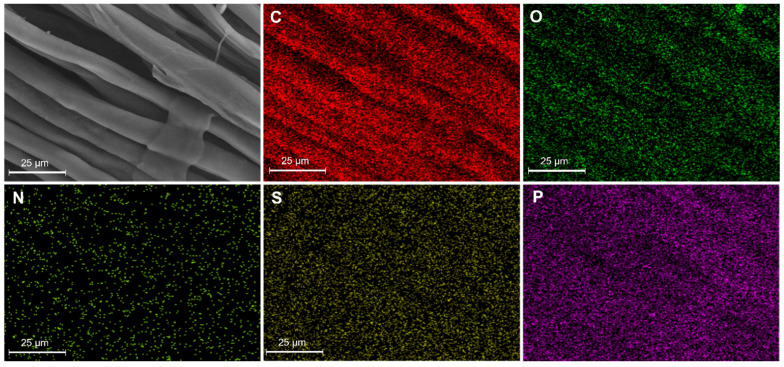
EDX analysis of COT/M-PCASS residues left by HFSTs. Distribution of carbon (C), oxygen (O), nitrogen (N), sulfur (S), and phosphorus (P).

The EDX analysis of the COT/M-PCASS residue after the HFSTs (Figure 7) showed a homogeneous distribution and fine dispersion of all elements, particularly nitrogen (N), sulfur (S), and phosphorous (P) in the investigated area. This suggested that both the phosphonate and disulfide groups are active in the condensed phase, in agreement with what previously reported [19,20,30].

#### 3.5.2. Vertical Flame Spread Tests

**Figure 8:**
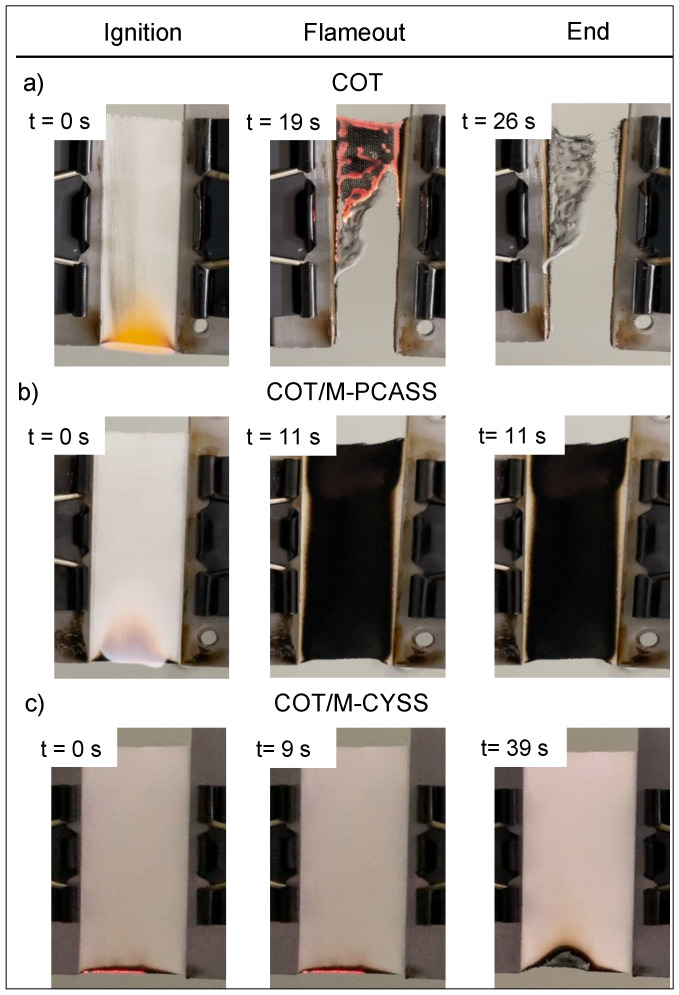
Snapshots of untreated cotton (**a**) and cotton fabrics treated with M-PCASS (**b**) and M-CYSS (**c**) at the ignition, flameout, and extinguishment steps during VFSTs.

VFSTs are more drastic than HFSTs due to the natural upright direction of the flame, which increases the contact area between the sample and the flame. For this reason, in VFSTs, untreated cotton burned faster than in HFSTs, evidently leaving no residues at the end of the test (Figure 8a). In VFSTs, cotton specimens were treated with M-PCASS and M-CYSS with a higher add-on (16%) compared to HFSTs (Table 4). At this add-on, while COT/M-CYSS (Figure 8c) extinguished the flame, leaving an RMF of 93%, COT/M-PCASS was not capable to suppress the flame (Figure 8b). However, thanks to the absence of an afterglow, the morphology of the residual fibers seemed to be intact in the whole sample.

#### 3.5.3. Oxygen-Consumption Cone Calorimetry Tests

**Figure 9:**
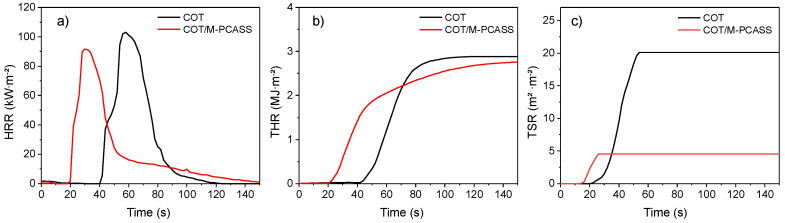
HRR (**a**), THR (**b**), and TSR (**c**) curves of untreated and M-PCASS-treated cotton fabrics.

To simulate a real fire scenario [27], untreated and M-PCASS-treated cotton samples were exposed to an irradiative heat flux of 35 kW∙m^−2^, similar to the heat generated in a developing fire [31], in an oxygen-consumption cone calorimeter. Combustion parameters such as time to ignition (TTI), heat release rate peak (pKHRR), total heat release (THR), and residual mass fraction (RMF) are reported in Table 5. Heat release rate (HRR) and total heat release (THR) curves are shown in Figure 9a and Figure 9b, respectively, as well.

Initially, untreated cotton vigorously burned and then, after flameout, occurring at circa 80 s, consumed completely due to afterglow (Figure 9a). By contrast, at an add-on as low as 8%, COT/M-PCASS burned more slowly, decreasing cotton pkHRR by 16% with no signs of afterglow. In details, the TTI value of COT/M-PCASS decreased by 24 s compared to untreated cotton (Table 5). However, in COT/M-PCASS, flameout occurred after about 20 s combustion in the presence of a flame, whereas flameout occurred after >30 s combustion in untreated cotton. After flameout, the COT/M-PCASS combustion slowly proceeded till the set end of the test (160 s) with no afterglow, leaving a final RMF of 10%, to be compared to an RMF < 1% for untreated cotton (Table 5). Furthermore, the THR of COT/M-PCASS was significantly lower than that of untreated cotton (Figure 9b).

In addition, the total smoke release during the initial non-flaming phase (TSR_(NFP)_) and during the flaming phase (TSR_(FP)_), CO and CO_2_ emission yields have been collected and reported in Table 6. The TSR curves of COT and COT/M-PCASS are shown in Figure 9c. M-PCASS coating strongly decreased the smoke released during cotton combustion in the absence and presence of a flame, as evidenced by the TSR_(NFP)_ and TSR_(FP)_ values (Table 6) and the TSR curve (Figure 9c). Noticeably, CO_2_ emissions from cotton combustion are halved thanks to M-PCASS. As regards CO emission, the values of CO yield collected by the analyzer were negligible for both untreated and treated cotton fabric.

In previous work [20], it should be observed that M-CYSS turned out to be more efficient than M-PCASS in reducing cotton pkHRR (50 vs. 16% for COT/M-PCASS and COT/M-CYSS, respectively). On the other hand, in this work, it was demonstrated that M-PCASS is more efficient in protecting cotton, since the final residue of COT/M-PCASS doubles with respect to that of COT/M-CYSS (10.0 vs. 5.0% for COT/M-PCASS and COT/M-CYSS, respectively). In addition, the reduction of CO_2_ emission is much more significant (50% vs. 10% for COT/M-PCASS and COT/M-CYSS, respectively).

## 4. Conclusions

Several polyamidoamines that have been shown to be able to protect cotton from combustion in horizontal flame spread tests by sensitizing cotton to thermal decomposition and promoting its intumescence in the temperature range of 350–450 °C. In addition, disulfide-containing polyamidoamines, including M-CYSS, obtained by the reaction of *L*-cystine with *N*,*N*′-methylenebisacrylamide, proved active as a flame retardant of cotton also in the severe vertical flame spread tests [20], possibly due to their ability to act as radical scavengers. Additionally, several phosphonates have been shown to be highly effective as flame retardants for cotton [6], due to the ability to enhance char formation at high temperatures [15]. Their mechanism of action was explained by hypothesizing that they acted in the condensed phase favoring the dehydration of cellulose rather than depolymerization once converted into the acid form.

The aim of this work was to ascertain whether the introduction of phosphonate groups in disulfide-containing PAAs could increase their already high efficacy as flame retardants for cotton. To this purpose, *N*,*N*′-methylenebisacrylamide was reacted with tetraethyl(((disulfanediylbis(ethane-2,1-diyl))bis(azanediyl))bis(ethane-2,1-diyl))bis(phosphonate), coded PCASS, to give a new polyamidoamine, coded M-PCASS, bearing two diethylphosphonate groups and a disulfide group per repeat unit. It should be noted that this is the first time ever that polyamidoamines-containing phosphonate groups have been investigated as flame retardants.

M-PCASS was applied as a coating on cotton fabrics and its flame-retardant efficacy, evaluated by several combustion tests, choosing the polyamidoamine coded M-CYSS, which contains disulfide groups but no phosphonate groups, as a benchmark. In horizontal flame spread tests, both M-PCASS and M-CYSS suppressed cotton combustion, but the former was more efficient than the latter. Specifically, the minimum M-PCASS add-on required for extinguishment was 8%, to be compared with a minimum M-CYSS add-on of 12%. Moreover, in contrast to what was observed in the M-CYSS-coated cotton, no afterglow was observed in the M-PCASS-coated cotton. On the other hand, in vertical flame spread tests, M-CYSS extinguished cotton combustion at a 16% add-on, whereas M-PCASS did not.

Morphological analysis of the burnt areas of M-PCASS-treated cotton fabrics subjected to horizontal flame spread tests demonstrated that the polyamidoamine coating bonded the cotton fibers upon melting.

Oxygen-consumption cone calorimetry tests showed that M-PCASS decreased the HRR peak of cotton by 16%, in addition to generating an RMF of 10.0%, to be compared with the RMF < 1% of untreated cotton. Noticeably, the M-PCASS coating was able to decrease the smoke release by 83% and, in addition, CO_2_ emissions were halved.

The set of results collected in this work allows us to formulate initial interesting considerations regarding the benefits deriving from the introduction of phosphonate groups in the repeat units of disulfide-containing polyamidoamines. It may be observed that they gave rise, overall, to appreciable improvements in horizontal flame spread tests, although they could not suppress cotton combustion in the severe vertical flame spread tests. Additionally, they induced a dramatic reduction in gas and smoke release, which endowed them with a potential as flame retardant in specific fire scenarios where the fume and gas abatement was a stringent requirement. This points to the suitability of carrying out further studies on the flame-retardant properties of polyamidoamines containing phosphonate groups with different chemical structures and architectures.

## Figures and Tables

**Table 1:** Thermal data of PAAs in nitrogen and air by thermogravimetric analysis.

Sample	T_onset10%_ ^1^ (°C)	T_max1_ ^2^ (°C)	T_max2_ ^3^ (°C)	RMF_800_ ^4^ (%)
*Nitrogen*				
M-PCASS	149	243	-	25
M-CYSS	157	246	-	0
*Air*				
M-PCASS	154	238	600	17
M-CYSS	154	242	449	23

^1^ Onset decomposition temperature at 10% weight loss. ^2^ First temperature at maximum weight loss rate. ^3^ Second temperature at maximum weight loss rate. ^4^ Residual mass fraction at 800 °C.

**Table 2:** Thermal data of COT/PAA samples in nitrogen and air by thermogravimetric analysis.

Sample	T_onset10%_ ^1^ (°C)	T_max1_ ^2^ (°C)	T_max2_ ^3^ (°C)	RMF_800_ ^4^ (%)
*Nitrogen*				
COT	330	365	-	5
COT/M-PCASS	292	340	-	29
COT/M-CYSS	250	302	-	23
*Air*				
COT	328	345	480	0
COT/M-PCASS	256	301	509	2
COT/M-CYSS	256	299	507	6

^1^ Onset decomposition temperature at 10% weight loss. ^2^ First temperature at maximum weight loss rate. ^3^ Second temperature at maximum weight loss rate. ^4^ Residual mass fraction at 800 °C.

**Table 3:** Combustion data of untreated cotton and PAA-treated cotton fabrics in HFSTs.

Sample	Add-on ^1^ (%)	Combustion Time ^2^ (s)	Afterglow	Extinguishment	RMF ^3^ (%)
COT	-	53	YES	NO	<1
COT/M-PCASS	8	34	NO	YES	81
COT/M-CYSS	12	19	YES	YES	93

^1^ Add-on ± 0.5%. ^2^ Combustion time ± 1 s. ^3^ RMF ± 1.0%.

**Table 4:** Combustion data of untreated cotton and PAA-treated cotton in VFSTs.

Sample	Add-on ^1^ (%)	Combustion Time ^2^ (s)	Afterglow	Extinguishment	RMF ^3^ (%)
COT	-	53	YES	NO	<1
COT/M-PCASS	16	11	NO	NO	45
COT/M-CYSS	16	20	YES	YES	96

^1^ Add-on ± 0.5%. ^2^ Combustion time ± 1 s. ^3^ RMF ± 1.0%.

**Table 5:** Combustion data of untreated and M-PCASS-treated cotton fabrics by oxygen-consumption cone calorimetry.

Sample	TTI ^1^ (s)	pkHRR ^2^ (kW∙m^−2^) (Reduction, %)	RMF ^3^ (%)
COT	45 ± 5	109 ± 7	<1
COT/M-PCASS	21 ± 1	92 ± 2 (−16)	10.0 ± 0.1

^1^ TTI: Time to ignition. ^2^ pKHRR: Heat release rate peak. ^3^ Residual mass fraction.

**Table 6:** Cone calorimetry smoke and gas data of untreated and PAA-treated cotton fabrics by oxygen consumption cone calorimetry.

Sample	TSR ^1^ (m^2^∙m^−2^)	TSR_(NFP)_ ^2^ (m^2^∙m^−2^) (Reduction, %)	TSR_(FP)_ ^3^ (m^2^∙m^−2^) (Reduction, %)	(CO) (kg∙kg^−1^)	(CO_2_) (kg∙kg^−1^)
COT	20.0 ± 0.5	15.8 ± 1.2	4.0 ± 0.7	0.0001 ± 0.0001	0.0055 ± 0.0025
COT/M-PCASS	4.5 ± 0.4	2.7 ± 0.7 (−83)	1.8 ± 1.0 (−55)	0.0001 ± 0	0.0025 ± 0

^1^ TSR: Total smoke release. ^2^ TSR_(NFP)_: Total smoke release during non-flaming phase. ^3^ TSR_(FP)_: Total smoke release during flaming phase.

## Data Availability

Not applicable.

## Supplementary Materials

The following supporting information can be downloaded at: https://www.mdpi.com/article/10.3390/polym15081869/s1, Figure S1: ^1^H-NMR spectrum of PCASS monomer; Figure S2: ^31^P-NMR spectrum of PCASS monomer; Figure S3: ^1^H-NMR spectrum of M-PCASS; Figure S4: ^1^H-NMR spectrum of M-CYSS; Figure S5: FT-IR/ATR spectrum of M-PCASS; Figure S6: FT-IR/ATR spectrum of M-CYSS; Figure S7: FT-IR/ATR spectra of untreated cotton (COT), COT/M-PCASS, and COT/M-CYSS.
