# Supplementary material for: On the Suitability of Phosphonate-Containing Polyamidoamines as Cotton Flame Retardants
Source: Polymers (Basel). 2023 Apr 13;15(8):1869. doi: 10.3390/polym15081869 (PMC10144353; doi:10.3390/polym15081869)
Supplement: Supplementary file 1 [file polymers-15-01869-s001.zip › polymers-2302664-supplementary.pdf]

Supplementary Materials

On the suitability of phosphonate-containing polyamidoamines as cotton flame retardants

Alessandro Beduini[1], Domenico Albanese[1], Federico Carosio[2], Amedea Manfredi[1], Elisabetta Ranucci[1], Paolo Ferruti[1], Jenny Alongi[1*]

[1] Dipartimento di Chimica, Università degli Studi di Milano, Via C. Golgi 19, 20133 Milan, Italy.
[2] Dipartimento di Dipartimento di Scienza Applicata e Tecnologia, Politecnico di Torino, Alessandria campus, Via T. Michel 5, 15121 Alessandria, Italy.

* Correspondence: jenny.alongi@unimi.it; Tel.: +39 0250314108

Pages S1-S6

Figures S1 and S2: ^{1}H- and ^{31}P-NMR spectra of PCASS monomer.

Figures S3 and S4: ^{1}H-NMR spectra of M-PCASS and M-CYSS.

Figures S5 and S6: FT-IR/ATR spectra of M-PCASS and M-CYSS.

Figure S7: FT-IR/ATR spectra of untreated cotton (COT), COT/M-PCASS and COT/M-CYSS.

NMR characterizations

The chemical structure of phosphorus-sulfur monomer, coded as PSSP, was assessed by ^{1}H-, ^{13}C- and ^{31}P-Nuclear Magnetic Resonance (^{1}H-NMR and ^{31}P-NMR, respectively) collecting spectra in D_2O at pH 4.0 and at 25 °C using a Brüker Advance DPX-400 NMR spectrometer (Milan, Italy) operating at 400.13 MHz and in $CDCl_3$ at 25 °C using a Brüker AC 300 spectrometer (Milan, Italy) operating at 75.3 MHz for ^{13}C-NMR and 121.5 MHz for ^{31}P-NMR. Chemical shifts were reported by using $CHCl_3$ (77.0 for ^{13}C-NMR) and 85% H_3PO_4 (0 ppm for ^{31}P-NMR) as external standards.

The chemical structure of PAAs, M-PCASS and M-CYSS, was assessed by ^{1}H-NMR, collecting spectra in D_2O at pH 4.0 and at 25 °C using a Bruker Advance DPX-400 NMR spectrometer (Milan, Italy) operating at 400.13 MHz. Parameters: scan number 32, relaxation delay, *d1*, 10.0 s, receiver gain automatically measured and set by the instrument.

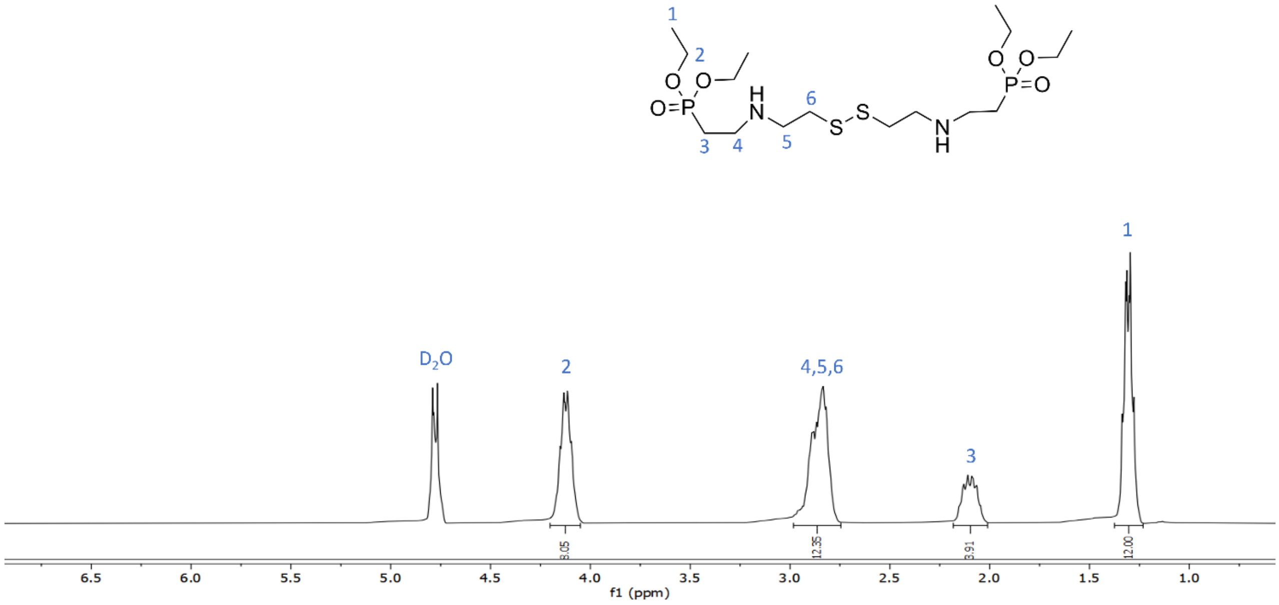

Figure S1. ^{1}H-NMR spectrum of PCASS monomer.

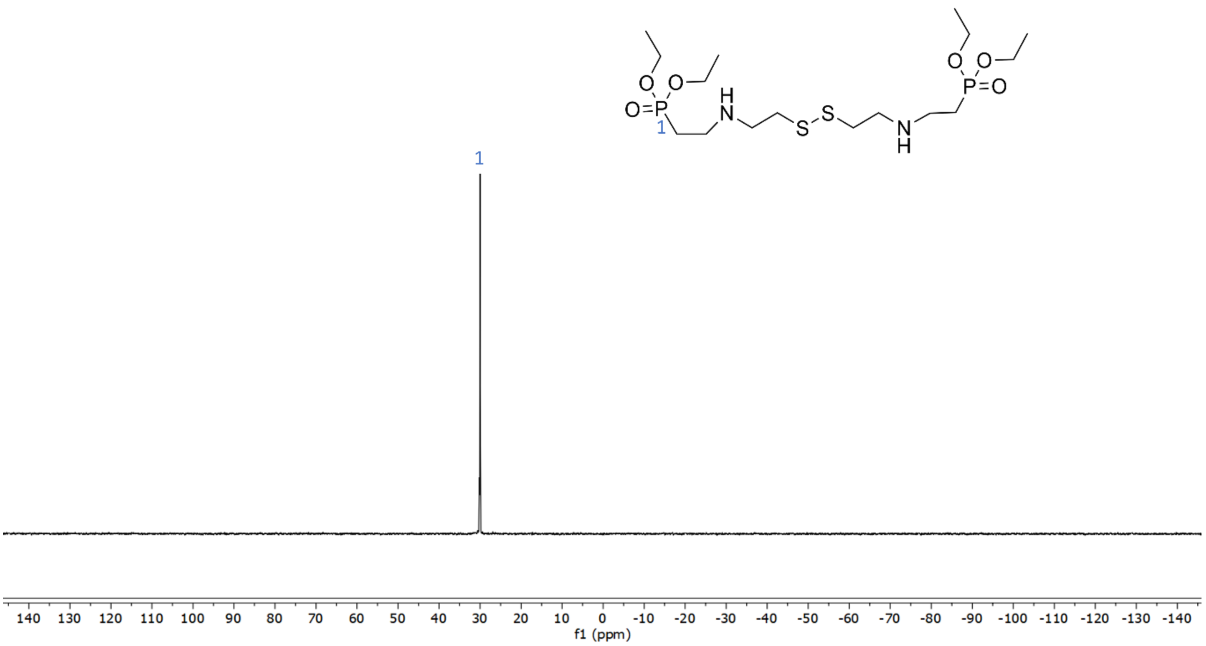

Figure S2. ^{31}P-NMR spectrum of PCASS monomer.

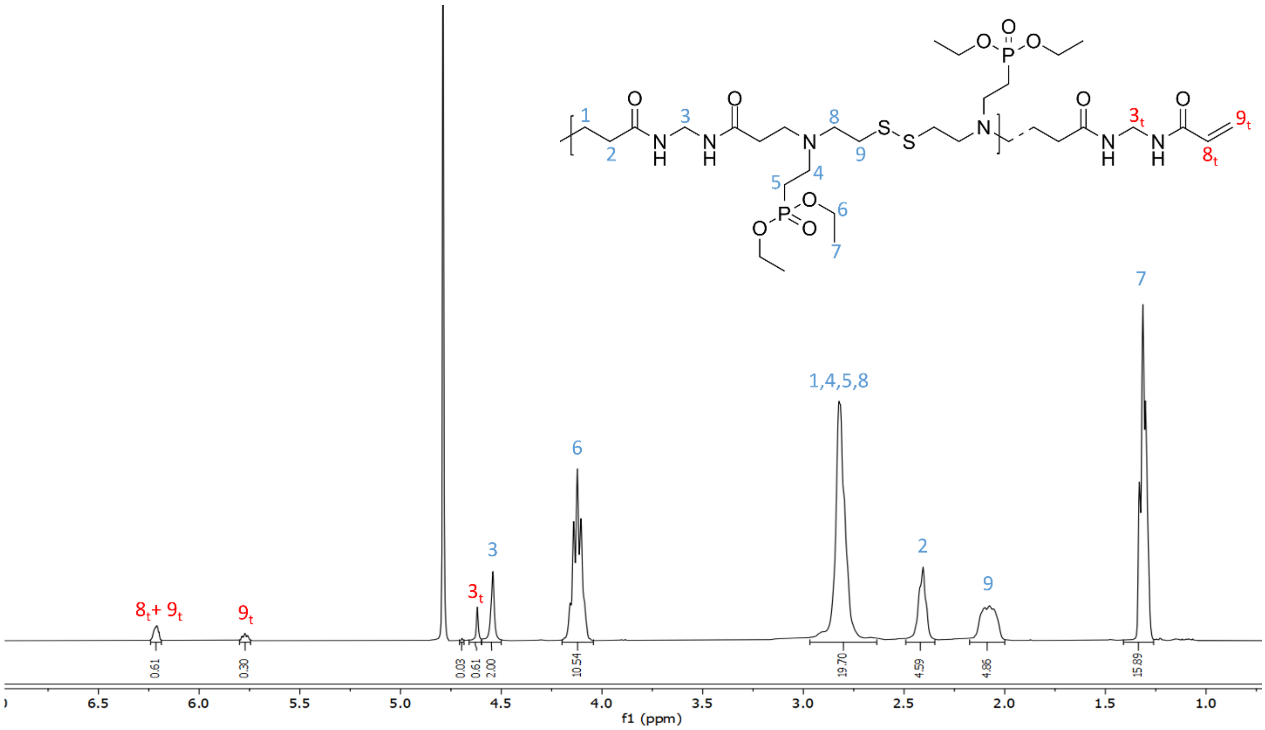

Figure S3. ^{1}H-NMR spectrum of M-PCASS.

$\bar{X}_n$ value was calculated as follows: $\bar{X}_n = \frac{1+\frac{H_3}{H_3+H_{3t}}}{1-\frac{H_3}{H_3+H_{3t}}}$

where H_3 is the integral of the H atom of the internal repeat unit and H_{3t} is the integral of the H atom of the terminal unit.

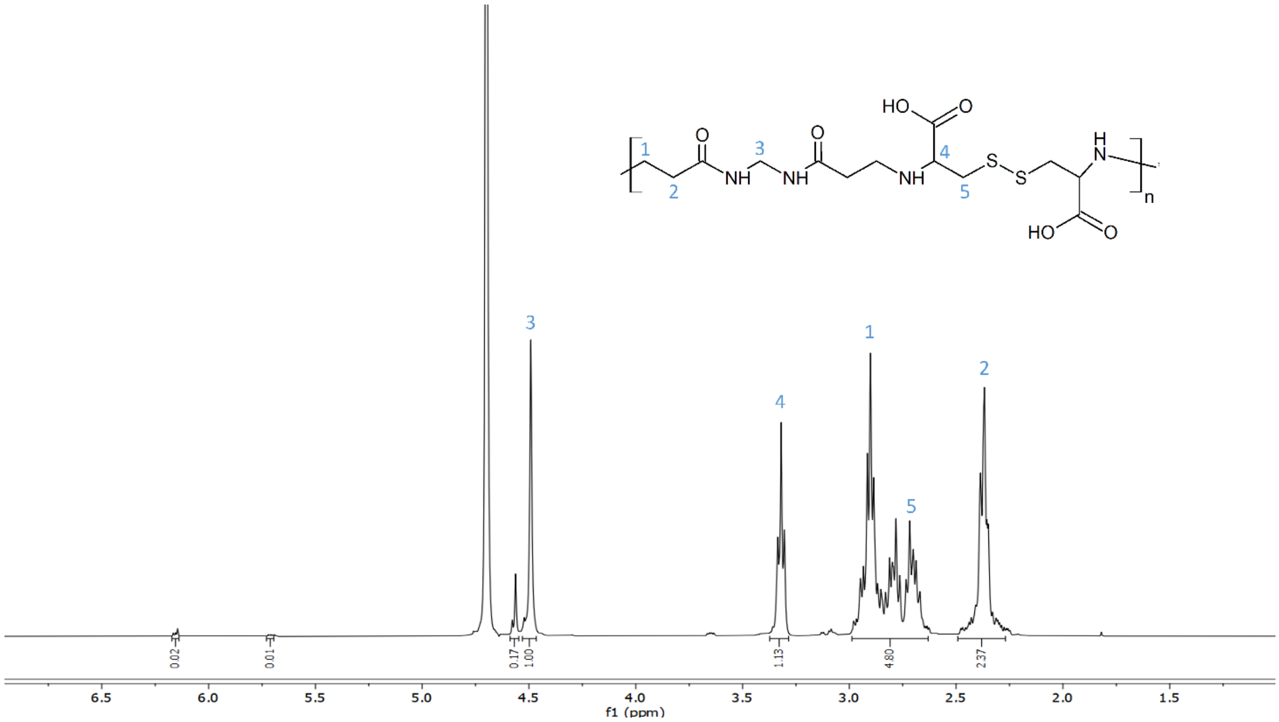

Figure S4. ^{1}H-NMR spectrum of M-CYSS.

FT-IR/ATR characterization

M-PCASS, M-CYSS, untreated cotton (COT), COT/M-PCASS and COT/M-CYSS were analyzed by attenuated total reflectance (ATR) Fourier transform infrared spectroscopy (FT-IR). FT-IR/ATR spectra were recorded at room temperature, in the 4000 - 500 cm^{-1} wavenumber range, with 128 scans and 4 cm^{-1} resolution using a Jasco FT/IR-4600 spectrometer (Lecco, Italy), equipped with a diamond crystal (penetration depth 1.66 mm).

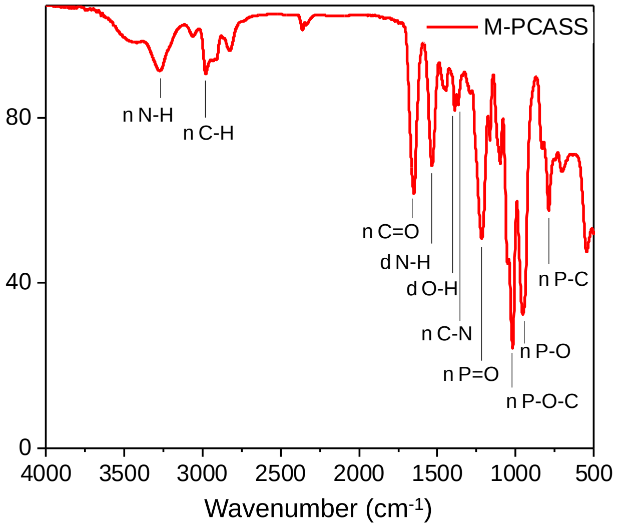

Figure S5. FT-IR/ATR spectrum of M-PCASS.

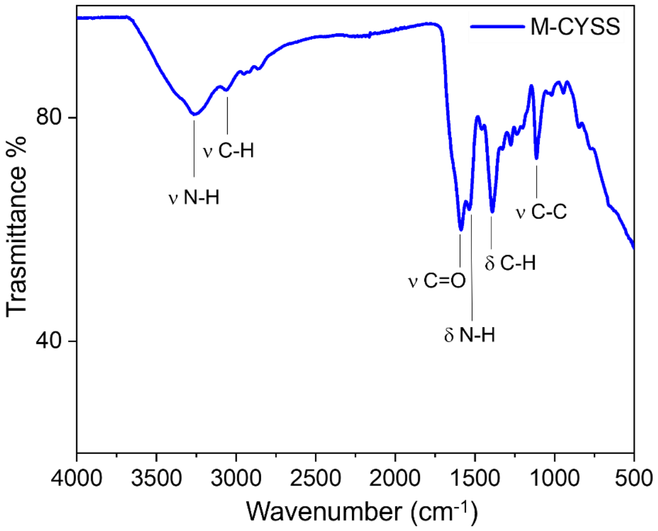

Figure S6. FT-IR/ATR spectrum of M-CYSS.

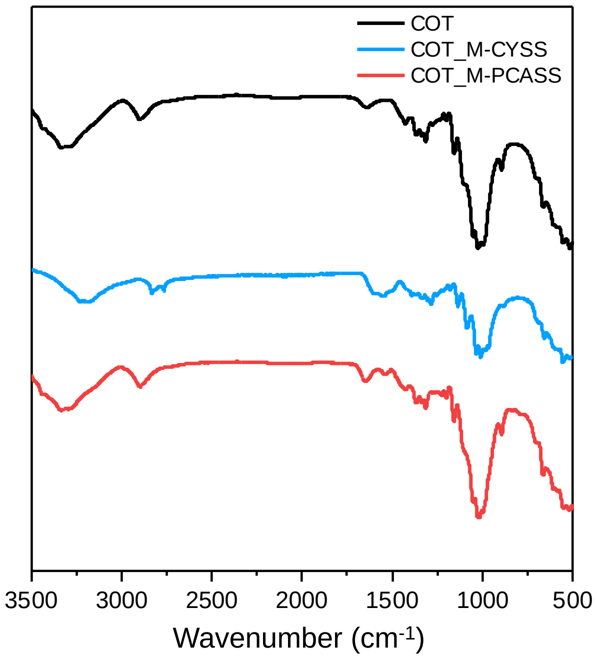

Figure S7. FT-IR/ATR spectra of untreated cotton (COT), COT/M-CYSS and COT/M-PCASS; diagnostic peaks were reported.
